# The International Conference on Intelligent Biology and Medicine (ICIBM) 2016: from big data to big analytical tools

**DOI:** 10.1186/s12859-017-1797-3

**Published:** 2017-10-03

**Authors:** Zhandong Liu, W. Jim Zheng, Genevera I. Allen, Yin Liu, Jianhua Ruan, Zhongming Zhao

**Affiliations:** 1 Jan and Dan Duncan Neurological Research Institute, Texas Children’s Hospital, Houston, TX 77030 USA; 2 Department of Pediatrics-Neurology, Baylor College of Medicine, Houston, TX 77030 USA; 3 School of Biomedical Informatics, The University of Texas Health Science Center, Houston, TX 77030 USA; 4 Department of Statistics, Rice University, Houston, TX 77030 USA; 5 Department of Electrical and Computer Engineering, Rice University, Houston, TX 77030 USA; 6 Department of Neurobiology and Anatomy, The University of Texas Medical School at Houston, Houston, TX 77030 USA; 7 Department of Computer Science, The University of Texas at San Antonio, San Antonio, TX 78249 USA; 8 Center for Precision Health, School of Biomedical Informatics, The University of Texas Health Science Center at Houston, Houston, TX 77030 USA; 9 Human Genetics Center, School of Public Health, The University of Texas Health Science Center at Houston, Houston, TX 77030 USA

## Abstract

The 2016 International Conference on Intelligent Biology and Medicine (ICIBM 2016) was held on December 8–10, 2016 in Houston, Texas, USA. ICIBM included eight scientific sessions, four tutorials, one poster session, four highlighted talks and four keynotes that covered topics on 3D genomics structural analysis, next generation sequencing (NGS) analysis, computational drug discovery, medical informatics, cancer genomics, and systems biology. Here, we present a summary of the nine research articles selected from ICIBM 2016 program for publishing in *BMC Bioinformatics.*

## Introduction

The 2016 International Conference on Intelligent Biology and Medicine (ICIBM 2016) provided a multidisciplinary forum for computational scientists and experimental biologists to share their most recent findings in the field of cancer genomics, systems biology, medical informatics, big data analytics and machine learning, among others. The conference was held on December 8–10, 2016 in Houston, Texas, USA. More than 150 researchers and students across the world attended the meeting. In this special issue, we have collected ten primary research articles focusing on new methods developed in the field of machine learning, genomics, and next generation sequencing (NGS) analysis.

In the first paper of this collection, Young et al. [1] developed a new unsupervised deep learning method to find low dimensional representations of cancer gene expression data. The estimated latent variables taken from the hidden layers of a deep-net provided novel insights into the mechanisms of tumorigenesis and patient survival. They discovered that the hidden layer representations encoded the information that was relevant to the clustering of glioblastoma samples and the survival of glioblastoma patients. This clustering also allowed them to uncover latent phenotype from the methylation data. In addition, model selection results provided a biologically plausible size for the first hidden layer. Understanding the biological relationships encoded in these hidden layer representations could lead to novel insights into cancer biology and treatment.

Philips et al. [2] used text mining algorithms to mine medical abstracts and identify new genes essential for cancer cell survival. The authors collected a corpus of 32,164 RNA interference abstracts from 10.5 million PubMed abstracts across various disciplines using database querying and text mining algorithms. Most of the top essential genes identified and extracted through these procedures are involved in the survival pathways and in various malignancies. Moreover, several of the top essential genes have not been previously implicated as essential oncogenes in the literature and could be novel targets on treating complex diseases and cancers.

In the next paper, Tang et al. [3] developed a new method, STRScan, that quantifies short tandem repeats (STRs) from whole-genome sequencing data. STRscan identifies k-mers from short NGS reads that are similar to input STR patterns. The authors then used a greedy seed-based algorithm to quantify the STRs. The authors tested their algorithm on whole genome sequencing data from the 1000 Genomes project and Venter’s genome. Their results demonstrated a 20% increase in identification of STRs compared to the existing approaches. The algorithm was also implemented in programming language C with an open-source license.

Non-canonical splicing is emerging as a new feature associated with a broad range of disease including cancer and neurological diseases. Bai et al. [4] developed a novel splice junction algorithm, Read-Split-Fly (RSF), to identify genome-wide non-canonically spliced regions. Preliminary results using RSF on the 70 ENCODE samples indicated that the presence of 5′ splicing site with U12-type signature is more frequent than U2-type in non-canonical junctions. The RSF algorithm will likely have a significant impact in the field by addressing the “gap in knowledge” involving undiscovered spliced sequences.

PennCNV is a highly-cited tool in the field of genomics. Lima et al. [5] extended this popular software to estimate copy number variations (CNVs) from whole genome sequencing data, by processing the mapping (BAM) files to extract coverage, representing log R ratio (LRR) of signal intensity, as well as B allele frequency (BAF) information. They tested the method using high quality sample NA12878 from the recently reported NIST database and ten simulated artificial samples with several CNVs spread along all chromosomes. The new method, PennCNV-Seq, can also be integrated in existing CNV calling pipelines to report accurately the number of copies in specific genomic regions.

RNA sequencing (RNA-seq), a high throughput technology that profiles gene expression, has been widely used for testing differential expression (DE) and more recently for testing differential alternative polyadenylation (APA). Liu et al. [6] extended and expanded the XBSeq BioConductor package yielding the XBSeq2 package. Specific major updates included alternative statistical testing and parameter estimation procedures, capacity to directly process alignment files and methods for testing differential APA, as well as major computational improvements that yield a faster package. The XBSeq2 package performs well on benchmarks compared to other approaches for testing DE and differential APA.

In the next paper, Tan et al. [7] proposed an expectation least squares (ELS) algorithm and binomial analysis of three-point gametes (BAT) for estimating gamete frequencies from F_2_ dominant and codominant genotype data, respectively. Using simulated and real datasets, ELS algorithm was able to accurately estimate frequencies of gametes and outperformed the EM algorithm in recovering true linkage maps in coupling and repulsive linkage phases. The ELS algorithm can be extended to search for accurate estimation of variables hidden in complex data. The BAT method had also high efficiency and fast speed in estimation of recombination fractions between codominant markers.

Li et al. [8] introduced a novel convolutional neural network (CNN) architecture for biomedical entity normalization, or linking entity mentions in text to those in a standard knowledge base. The procedure uses a rule-based approached followed by CNN modeling of semantic similarity that is used to yield a final ranking of entities. This approach achieves accuracies of 90.30% and 86.10% when evaluated on the ShARe/CLEF and NCBI datasets, respectively, which greatly improves upon the existing state-of-the-art rule-based baseline systems.

Scaffold proteins play a critical role in various biological signaling processes. While many databases were documented to link to the signaling pathways, few databases are devoted to the scaffold proteins that medicate signal transduction. Here, Han et al. [9] developed a user-friendly interface database, ScaPD, to curate computationally predicted, experimentally validated scaffold proteins and associated signaling pathways. It currently contains 273 scaffold proteins and 1118 associated signaling pathways. The database allows users to search, navigate and download the scaffold protein-mediated signaling networks. Manually curated and predicted scaffold protein data form a foundation for further investigation of scaffold proteins and signal transduction. ScaPD will also be a valuable resource for understanding how individual signaling pathways are regulated.

## References

[1] Young JD, Cai C, Lu X. Unsupervised deep learning reveals Prognostically relevant subtypes of Glioblastoma. BMC Bioinformatics. 2017;18(Suppl 11): doi:10.1186/s12859-017-1798-2. 10.1186/s12859-017-1798-2 PMC5629551 28984190

[2] Philips S, Wu HY, Li L. Using machine learning algorithms to identify genes essential for cell survival. BMC Bioinformatics. 2017;18(Suppl 11): doi:10.1186/s12859-017-1799-1. 10.1186/s12859-017-1799-1 PMC5629548 28984184

[3] Tang H, Nzabarushimanaa E. STRScan: targeted profiling of short tandem repeats in whole-genome sequencing data. BMC Bioinformatics. 2017;18(Suppl 11): doi:10.1186/s12859-017-1800-z. 10.1186/s12859-017-1800-z PMC5629557 28984185

[4] Bai Y, Kinne J, Ding L, Rath EC, Cox A, Naidu SD, Deng Y. Identification of genome-wide non-canonical spliced regions and analysis of biological functions for spliced sequences using read-split-fly. BMC Bioinformatics. 2017;18(Suppl 11): doi:10.1186/s12859-017-1801-y. 10.1186/s12859-017-1801-y PMC5629565 28984182

[5] Lima LA, Wang K. PennCNV in whole-genome sequencing data. BMC Bioinformatics. 2017;18(Suppl 11): doi:10.1186/s12859-017-1802-x. 10.1186/s12859-017-1802-x PMC5629549 28984186

[6] Liu Y, Wu P, Zhou J, Johnson-Pais TL, Lai Z, Rodriguez R, Chen Y. XBSeq2: a fast and accurate quantification of differential expression and differential polyadenylation A. BMC Bioinformatics. 2017;18(Suppl 11): doi:10.1186/s12859-017-1803-9. 10.1186/s12859-017-1803-9 PMC5629564 28984183

[7] Tan YD, Zhang XH, Mo Q. New statistical methods for estimation of recombination fractions in F2 population. BMC Bioinformatics. 2017;18(Suppl 11): doi:10.1186/s12859-017-1804-8. 10.1186/s12859-017-1804-8 PMC5629630 28984187

[8] Li H, Chen Q, Tang B, Wang X, Wang Z, Xu H, Wang B, Huang D. CNN-based ranking for biomedical entity normalization. BMC Bioinformatics. 2017;18(Suppl 11): doi:10.1186/s12859-017-1805-7. 10.1186/s12859-017-1805-7 PMC5629610 28984180

[9] Han X, Wang J, Wang J, Liu S, Hu J, Zhu H, Qian J. ScaPD: a database for human scaffold proteins. BMC Bioinformatics. 2017;18(Suppl 11): doi:10.1186/s12859-017-1806-6. 10.1186/s12859-017-1806-6 PMC5629588 28984188

